# Utilization of awake craniotomy for supra-tentorial tumor resection during pregnancy: A technique useful for fetal-maternal wellbeing

**DOI:** 10.12669/pjms.36.2.1853

**Published:** 2020

**Authors:** Dileep Kumar, Sheema Siraj, Khalid Ahsan, Faraz Shafiq

**Affiliations:** 1 Dileep Kumar, FCPS. Department of Anesthesiology, The Aga Khan University, Stadium road, Karachi, Pakistan; 2 Sheema Siraj, FCPS. Department of Anesthesiology, The Aga Khan University, Stadium road, Karachi, Pakistan; 3 Khalid Ahsan, FCPS. Department of Anesthesiology, The Aga Khan University, Stadium road, Karachi, Pakistan; 4 Faraz Shafiq, FCPS. Department of Anesthesiology, The Aga Khan University, Stadium road, Karachi, Pakistan

**Keywords:** Pregnancy, Supratentorial, Craniotomy and Wakefulness

## Abstract

Meningioma is the benign tumor that can also occurs during pregnancy. We are reporting a case of 29 years, 13th weeks pregnant lady, who underwent supratentorial craniotomy using awake through out approach. The case report highlights the challenges we faced during anaesthetic management, which includes psychological preparation, institution of scalp block and successful neurological monitoring. Technique proven to be useful considering pregnancy related physiological and tumor related pathological changes, the impact of which lies directly on maternal & fetal wellbeing.

## INTRODUCTION

Meningioma rarely presents in pregnancy. Once appeared, their growth rate is relatively increased.^1^ Neurosurgical intervention is usually required when patient develops instability, worsening of cranial pathology on imaging studies and at times during pregnancy.^2^ The choice between general anaesthesia (GA) versus awake craniotomy (AC) is based on location of tumor and need of intraoperative neurological monitoring. However, patient’s motivation, available expertise and fetal-maternal wellbeing are additional factors which needs to be considered in this scenario. We are reporting a successful management of gross total resection of meningioma in pregnant lady using AC. The case report highlights the importance of awake throughout (AT) approach in terms of patient’s acceptance, neurological outcome and fetal-maternal well-being.

## CASE

Our patient was 29-year Asian female. She was gravida 3 and para 2+0, weighing 53 Kg and height of 153 cm. She presented at 13^th^ weeks of gestation with complaint of “*Left eye blurred vision”* since seven months. Past medical and obstetric history was insignificant. She noticed aggravation of her symptoms since 4th week of gestation. The Magnetic resonance imaging (MRI) brain revealed broad based extra-axial lesion in right frontal region causing marked mass effect on adjacent brain parenchyma. The provisional diagnosis was *“Right frontal meningioma”*. The neurosurgical team selected this patient for AC. Primary anaesthetist did preoperative assessment and psychological counseling. The pre-operative GCS was 15, and she had no neurological deficits except left sided blurred vision. Her hemoglobin was 11.6g/dl, while hematocrit of 35.1%, for which she was labeled American Society of Anesthesiologist (ASA) grade II. The pelvic ultrasound was evident for alive 13^th^ weeks fetus. Obstetric monitored fetal heart sounds preoperatively, and administered cyclogest 800 mg rectally as a tocolytic agent. Routine ASA specified monitoring including EKG, pulse oximeter was done. Left radial artery cannulation was done for continuous blood pressure monitoring. The scalp block was instituted to provide intraoperative analgesia. This was done using anatomical landmark technique.^3^ The supplemental oxygen at two liters per minute via nasal prongs with inbuilt EtCO2 monitoring was applied. Once the effectiveness of scalp block was confirmed the patient’s head was fixed on Mayfield frame. Supra-tentorial craniotomy was performed. Patient remained comfortable and co-operative through out the procedure. Intraoperative neurological assessment was done for contralateral motor functions in both upper & lower limb. The surgery lasted for four hours during which gross resection of tumor was done. Estimated blood loss was the 600 ml. After the completion of surgery, patient was shifted to post anaesthesia care unit (PACU) with stable hemodynamics. The postoperative analgesia was provided using intermittent doses of Paracetamol and Tramadol. Post procedure obstetric review ensured fetus viability as evident by fetal heart sounds. Overall patient remained fairly stable and was discharged to home on 3rd postoperative day. Her follow up after one week revealed complete resolution of symptoms. The provisional diagnosis of meningioma was confirmed on histopathology.

## DISCUSSION

Meningioma is one of the most common benign tumors that occur during pregnancy. Its occurrence during pregnancy is not different from adult population comprising about 20% of all brain tumors, with an incidence of 5-6 per 100,000 pregnancies.^4^ However, studies have shown relationship between tumor growth and hormonal changes associated with pregnancy. These hormonal changes may be the reason of flaring up of symptoms during pregnancy.^5^ Neurosurgical intervention during pregnancy means massive alteration in patient’s physiology. One need to understand the physiological changes associated with pregnancy, and pathological consequences of tumor on cerebral physiology. Besides, an impact of anaesthetic technique on feto-maternal circulation needs to be considered. The use of AC in this scenario is logical not only to avoid GA and harmful effects of anaesthetic medications, but it also enhances recovery. The corner stone of anaesthetic management is preoperative psychological preparation and assessment of willingness from patient. The involvement of primary anaesthetist is helpful not only in reducing the anxiety but also for rapport building.

The additional challenge that we faced was her language barrier status. Preoperative counseling of patient was done with the help of translator that helped her in clear understanding about technique, possible discomforts and the need of being awake. Patient remained hemodynamically stable and pain free during the procedure. Besides, intraoperative neurological monitoring was done successfully which was helpful in doing gross total resection. The AT approach is routine at our health care setup. The technique has proven to be safe, very low complication rates including the conversion to GA. Similarly, it’s been used patients having variable socioeconomic and educational backgrounds, as well as having different co-morbidities.6 The success of this in pregnancy is addition to its applicability. Though studies have shown some association with preoperative anxiety and postoperative pain in patients recently diagnosed to have brain tumours.^7^ However, preoperative psychological preparation and perioperative support throughout the procedure is important to make this technique successful. This is evident by the fact that most patients have no recall about the intraoperative discomfort and declare better satisfaction postoperatively.^8^ Postoperative review of our patient also showed better satisfaction with the technique.

## CONCLUSION

Awake Through (AT) approach is logical and safe in pregnant patients requiring tumor resection during pregnancy. Anaesthetic management includes extensive preoperative psychological preparation and intraoperative use of scalp block. The technique provides hemodynamic stability, facilitates intraoperative neurological monitoring, tumor resection and improving fetal-maternal outcome.

### Authors’ Contributions:

**DK:** Preoperative preparation, perioperative care, initial write up.

**SS:** Case summary write up, consent from the patient, finalization of author Performa.

**KA:** Supervision during the clinical care being the credential for service.

**FS:** Literature search, Discussion, Proof reading, submission, is responsible for integrity of research.
